# Investigation of biological effects of HEMA in 3D-organotypic co-culture models of normal and malignant oral keratinocytes

**DOI:** 10.1080/26415275.2023.2234400

**Published:** 2023-07-13

**Authors:** Sunita Sharma, Qalbi Khan, Olaf Joseph Franciscus Schreurs, Dipak Sapkota, Jan Tore Samuelsen

**Affiliations:** a Nordic Institute of Dental Materials, Oslo, Norway; b Christiania Dental Clinic, Malo Dental, Oslo, Norway; c Department of Oral Biology, University of Oslo, Oslo, Blindern, Norway

**Keywords:** Oral cells, 2-hydroxethyl methacrylate, keratinocytes, dental materials

## Abstract

Several *in vitro* studies utilizing 2-dimensional (2D) cell culture systems have linked 2-hydroxyethyl methacrylate (HEMA) with cytotoxic effects in oral mucosa and dental pulp cells. Although such studies are invaluable in dissecting the cellular and molecular effects of HEMA, there is a growing interest in the utilization of appropriate 3-dimensional (3D) models that mimic the structure of oral mucosa. Using a previously characterized 3D-organotypic co-culture model, this study aimed to investigate the cellular and molecular effects of HEMA on a 3D-co-culture model consisting of primary normal oral keratinocyte (NOK) grown directly on top of collagen I gel containing primary oral fibroblasts (NOF). The second aim was to examine the suitability of a 3D-co-culture system consisting of oral squamous cell carcinoma (OSCC) cells as a model system to investigate the biological effects of HEMA. We demonstrated that HEMA treatment led to reduced viability of NOK, NOF and OSCC-cell lines in 2D-culture. The keratinocytes in 3D-co-cultures of NOK and OSCC-cells reacted similarly with respect to cell proliferation and activation of autophagy flux, to HEMA treatment. Nevertheless, NOK was found to be more susceptible to apoptosis following HEMA treatment than OSCC in 3D-co-cultures. These results indicate that 3D-organotypic co-cultures of NOK might represent an appropriate model system for the investigation of the biological effects of HEMA and other dental biomaterials. Given the challenges in obtaining primary cultures of NOK and issues associated with their rapid differentiation in culture, the possible use of OSCC cells as an alternative to NOK for 3D models represents an area for future research.

## 1. Introduction

Resin-based dental materials, based on the polymerization of organic methacrylate monomers and co-monomers like 2-hydroxethyl methacrylate (HEMA), are an integral part of modern dentistry. These materials are extensively used in various dental applications, such as restoration and adhesion procedures. Despite the advancements in curing/polymerization techniques, complete polymerization of these materials is never achieved *in situ*, and leaching/release of unpolymerized HEMA and other monomers from resin-based dental materials occurs in the oral cavity [1,2].

Several *in vitro* studies have linked HEMA with cytotoxic effects leading to suppression of cell proliferation and induction of apoptosis [3–6]. These effects are considered to be mediated by HEMA-induced depletion of cellular glutathione reserves and subsequent oxidative stress caused by imbalances in the production of reactive oxygen species (ROS) [7,8]. Nevertheless, our group and others have previously suggested the possible existence of glutathione and ROS-independent mechanisms related to HEMA-mediated cytotoxicity [9,10].

As one of the key defence mechanisms against cellular oxidative stress mediated by HEMA, cells activate Nrf2/ARE signalling pathway. Activation of the Nrf2/ARE signalling pathway leads to increased transcription of, among others, a number of genes coding for antioxidants and proteins involved in phase II biotransformation, and autophagy pathway including p62 (encoded by gene *SQSTM1*) [11–13]. Although the mechanisms involved in HEMA-induced activation of the Nrf2/ARE signalling pathway are not fully understood, both depletion of glutathione/oxidative stress-mediated (indirect) [12] and direct activation of molecules in the Nrf2-ARE transcriptional pathway associated with electrophilic properties of HEMA [13], have been suggested.

Although HEMA exposure and its possible biological effects have also been reported in cells in the skin and airways [14,15], oral mucosa, including the dental pulps, are considered to be the main targets of HEMA-mediated cellular effects associated with dental restorations with resin-based dental materials.

Oral mucosa consists of stratified squamous epithelium and underlying lamina propria. A continuous mutual interplay, not only mediated by the paracrine soluble factors but also by direct physical interactions between the epithelial keratinocytes and stromal fibroblasts, is considered to be important in proliferation, differentiation and shaping the overall structural and functional properties of the oral epithelium [16].

Several of the previous studies have utilized 2-dimensional (2D) cell culture model systems for the investigation of cellular effects of HEMA. Although such studies are invaluable in dissecting the cellular and molecular effects of HEMA on specific cell types, the clinical relevance of such models is not obvious. The cells grown in 2D culture are suggested to acquire a differential morphology and transcriptional profile related to key cellular functions such as proliferation, differentiation and migration as compared with the cells in 3D environment [17–19]. This underscores the utilization of appropriate 3D model systems mimicking the structure of oral mucosa to investigate the clinically relevant biological effects of HEMA on cells of the oral cavity. A recent study by Perduns et al. utilizing a 3D-model consisting of immortalized oral keratinocytes (OKF6/TERT2) and gingival fibroblasts, has demonstrated that HEMA could penetrate the layers of oral keratinocytes and induce a cellular oxidative defence response [20]. However, the 3D model lacked the direct physical interaction between the oral keratinocytes and fibroblasts as the cell types were separated by a porous membrane. Close physical proximity between the fibroblasts and overlying keratinocytes has been suggested to influence the migration of partially transformed oral keratinocytes in 3D co-culture models [21].

Difficulties in obtaining primary cultures of normal oral keratinocytes (NOK) and normal oral fibroblasts (NOF), and the establishment of three-dimensional organotypic culture models mimicking oral mucosa are considered to be the key challenges limiting the investigations of HEMA-mediated clinically relevant biological effects in the oral tissue. Among the primary oral cells, NOK cultures are notoriously difficult to establish and maintain for a longer period as they tend to differentiate after 3-4 passages in 2D cultures. In this scenario, the use of immortalized NOK or cells derived from oral squamous cell carcinoma (OSCC), which are easier to grow and maintain in culture, is gaining interest as a suitable alternative to NOK.

Using previously characterized 3D-organotypic co-culture model systems [22,23], the current study aimed to investigate the cellular and molecular effects of HEMA on a 3D-organotypic co-culture model consisting of primary NOK grown directly on top of collagen I gel containing primary fibroblasts. Another aim was to examine the suitability of a 3D-organotypic co-culture system consisting of OSCC-cells as a model system to investigate the biological effects of HEMA.

## 2. Materials and methods

### 2.1. Cell culture

Primary cell cultures were isolated from mucosal tissue obtained after third molar extractions as described recently [24]. In brief, the epithelial sheet and the connective tissue were separated by tissue digestion and cut into small pieces prior to cell isolation through enzymatic treatment. NOK from two donors (NOK1 and NOK2) were cultured in keratinocyte serum-free medium (GIBCO), supplemented with 25 μg/ml bovine pituitary extract (GIBCO), 1 ng/ml epidermal growth factor (GIBCO) and 1x antibiotic and antimycotic solution. NOF from three donors (NOF1, NOF2 and NOF3) were cultured in DMEM high glucose supplemented with 10% FBS, L-glutamine (Lonza) and 1× antibiotic and antimycotic solution. The squamous cell carcinoma-derived cell lines CaLH3 [25] and SCC25 [26] were grown in DMEM/F12 1:1 mixture (Gibco) supplemented with 10% FBS, 10 ng/ml epidermal growth factor, 0.4 µg/ml hydrocortisone, 0.05x ITS-A (Gibco), 50 µg/ml sodium L-ascorbate, 2% L-Glutamine as well as 1× antibiotic and antimycotic solution. Cells were grown in a humidified atmosphere of 5% CO2 in air at 37 °C. Non-specified cell culture media and solutions were obtained from Sigma (Merck). Isolation and the use of NOK and NOF in the current study were approved by the Regional Committees for Medical Research Ethics South East Norway (REK-2013/1818).

### 2.2. Cell viability assay

To investigate the viability of cells in 2D culture after treatment with HEMA, colorimetric assay resazurin (Cat no: 199303, Sigma-Aldrich) was used. Briefly, 5000 cells in 100 μL cell culture medium per well were seeded in a 96-well plate in 6 replicates and cultured for 24 h. The cells were subsequently treated with 8 mM or 4 mM HEMA and examined for cell viability after 6 or 24-h of treatment. Four hours before each measurement, resazurin (pre-diluted at a working concentration of 0.1 mg/ml in phosphate-buffered saline) was added to each well to a final concentration of 0.01 mg/mL. Following incubation for 4 h, the absorbance was measured at dual mode 560/590 nm with a scanning multiwell spectrophotometer (Epoch, BioTek Instruments). The results were analyzed in GraphPad Prism.

### 2.3. 3D-organotypic cultures

The 3-dimensional organotypic co-cultures were constructed (six technical replicates per experimental group) by growing either NOK (NOK1 and NOK2) or CaLH3 cells on top of a collagen biomatrix supplemented with fibroblasts, as described previously [22]. In brief, matrices containing collagen type I (Corning), DMEM (Gibco), 10% FBS (Gibco) and 2.5 × 10^5^ primary fibroblasts per matrix were prepared in 24 well plates and left to polymerize before covering with fibroblast medium. The next day, approximately 4x10^5^ NOK or CaLH3 were seeded on top of the matrix. Then, the matrices were transferred to a pre-wetted curved metal grid covered with a piece of lens paper, and placed in 6-well plates with OT medium with its level adjusted to keep the matrix in the air–liquid interface. The OT medium was identical to the medium used for the CaLH3 except for the FBS which was replaced by albumin for the organotypic with CaLH3 or linoleic acid – albumin for the organotypic with NOK, both with a final concentration of 0.1%.

3D-organotypic cultures with NOK and CaLH3 were treated with 4 mM HEMA on day 13 and on day 6, respectively. The selection of different time points for HEMA treatment in 3D-organotypic cultures with NOK and CaLH3 was based on the fact that NOK grows slower than OSCC both in 2D and in 3D cultures and therefore 3D-organotypic cultures of NOK take a longer time than that of OSCC to achieve a comparable epithelial thickness. After 6 or 24 h of treatment with HEMA, organotypic cultures were harvested. Briefly, one-half of each organotypic culture was fixed in 4% paraformaldehyde in PBS pH 7.4, 1/4^th^ was used for RNA and the remaining 1/4^th^ for protein isolation. Organotypic co-cultures were grown in a humidified atmosphere of 5% CO_2_ in air at 37 °C. Non-specified cell culture media and solutions were obtained from Sigma (Merck). 3D-organotypic cultures with NOK2) were used to validate *SQSTM1* mRNA expression after HEMA treatment.

### 2.4. RNA isolation, cDNA synthesis and qRT-PCR

Total RNA from organotypic cultures was isolated using the Total RNA Miniprep Kit (NEB #T2010) following the manufacturer’s instructions. Briefly, organotypic cultures were lysed in 500 µL of lysis buffer and after the removal of genomic DNA, total RNA was eluted in 50 µL of nuclease-free water. Two hundred nanograms of total RNA per sample was used to synthesize cDNA using LunaScript RT SuperMix Kit (NEB#E3010) in a reaction volume of 20 µL. qRT-PCR was performed in three technical replicates per sample using C1000 Touch Thermal cycler CFX96 Real-time system (Bio-Rad). Briefly, the 20 µL reaction volume consisted of 10 µL of 2x SsoAdvanced Universal Probes Supermix (#172-5280, BIO-RAD), 1 µL of cDNA, 1 µL of either *SQSTM1* (# qHsaCED0045925, BIO-RAD) or *GAPDH* (#qHsaCED0038674, BIO-RAD) SYBR Green assay and 8 µL of nuclease-free water. The relative gene expression was calculated using 2^–ΔΔCt^ method.

### 2.5. Immunohistochemical staining

Formalin-fixed, paraffin-embedded sections were baked at 60 °C for 2 h, deparaffinized and rehydrated following standard procedures. Endogenous peroxidase was blocked by 0.3% hydrogen peroxide in methanol for 30 min. Slides were placed in a Hellendahl jar containing TE buffer (10 mM Tris, 1 mM EDTA, 0.05% Tween-20, pH 9) and heat-induced epitope retrieval was performed for 15 min at 100 °C utilizing a decloaking chamber (Biocare Medical). After a cooling period of 20 min, slides were rinsed with water and equilibrated in phosphate-buffered saline (PBS) prior a 30 min blocking step with 5% normal serum matching the species of the secondary antibody. Sections were incubated overnight at 4 °C with the following primary antibodies: mouse monoclonal anti-Ki-67 (Agilent Cat# M7240, RRID:AB_2142367), rabbit polyclonal anti cleaved caspase-3 (Cell Signaling Technology Cat# 9661, RRID:AB_2341188) or Guinea pig polyclonal anti p62 protein (ProGen Cat# GP62-C, RRID:AB_2687531). After washing in PBS, the bound antibody was amplified by incubation with matched secondary antibodies for 1 h at 21 °C. For Ki-67 and cleaved caspase-3, the peroxidase labelled polymeric Dako EnVision + System (Agilent) was used, whereas p62 protein was amplified with biotinylated rabbit anti-Guinea pig IgG (Vector Laboratories) followed by a 30 min incubation with peroxidase-conjugated ABC reagent (Vector Laboratories). The peroxidase conjugates were visualized using 3,3′-diaminobenzidine as a substrate and subsequently intensified with 0.5% copper sulphate in saline. Finally, nuclei were counterstained with hematoxylin before dehydration and mounting of cover glass using Histokitt (Assistent, Karl Hecht).

### 2.6. Semi-quantitative evaluation of p62, Ki67 and cleaved Caspase-3 immunostaining in 3D-organotypic co-culture models

A minimum of three visual fields (region of interest, ROIs) from each 3D model replicate were randomly chosen (excluding the areas with distortion of tissue architecture related to the mechanical impact of tissue processing and epitope retrieval technique of the 3D models). Digital pictures for the selected ROIs were taken at 20X magnification using an Eclipse 90i microscope equipped with DS-Ri1 camera and NIS Elements F software (all Nikon Instruments). For the quantification of p62 immunostaining, the Image J IHC profiler plugin was used to calculate the positive color intensity, which will quantify the color intensity of an image. The results of quantification were converted into H-Score based on the following formula: H-Score = (% low positive × 1) + (% positive × 2) + (% high positive × 3), as described previously [27]. For Ki76, the positive and negative cells were counted at 20X magnification to determine the proliferation index.

For evaluation of Cleaved Caspase-3 positive cells, immuno-stained slides were scanned using a Pannoramic Midi II digital slide scanner at 20× magnification. The scans were analyzed with QuPath software (Version 0.3.2.). Cleaved Caspase-3 positivity was analyzed in three randomly selected areas from each slide containing a minimum of 500 cells (range: 724–3825 cells). The extreme ends of the sections and any areas containing artefacts were omitted, as well as the fibroblastic matrix and fully differentiated superficial keratinocyte layers. Counting was performed automatically using the positive cell detection tool. The analysis was blinded for the sample identity. One sample was excluded from the analysis due to suspicion of bacterial infection (NOK organotypic co-culture with HEMA exposure for 6 h), and another two were excluded due to lack of sufficient epithelium/number of keratinocytes (NOK organotypic co-culture with HEMA exposure for 6 and 24 h).

### 2.7. Statistical analyses

Statistical analyses were performed using GraphPad Prism for Windows (https://www.graphpad.com/; version 9.4.1). Unpaired student’s *t*-test was used to examine differences in means between two groups, while ANOVA with Tukey’s multiple comparison test was used for the comparison of means between three groups. Data are expressed as mean ± standard deviation (SD). A *p*-value of <0.05 was considered statistically significant.

## 3. Results

### 3.1. HEMA treatment significantly reduced the viability of NOK, NOF and OSCC cells in 2D monolayer cultures

**Figure 1.**
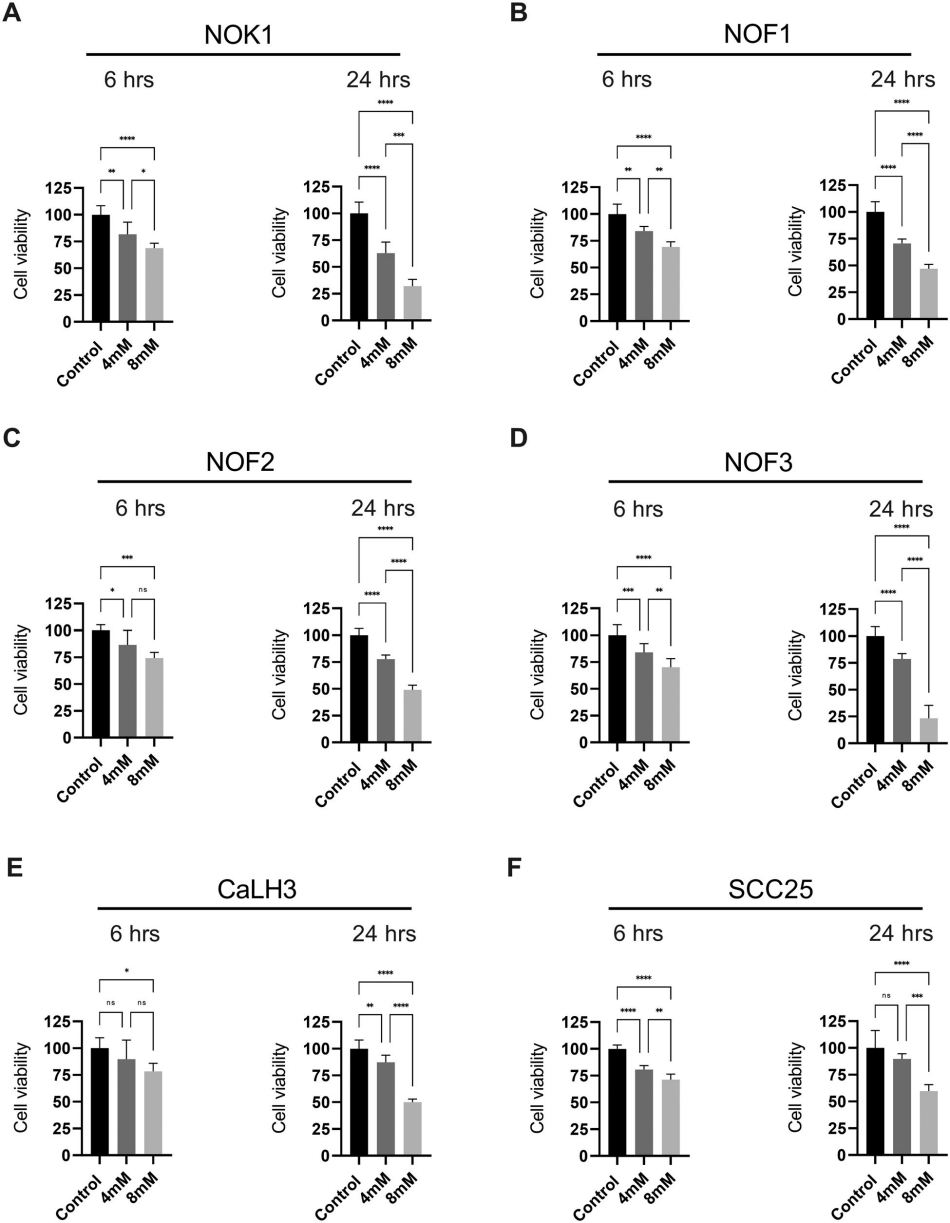
Reduction in the viability of NOK, NOF and OSCC cells in 2D monolayer cultures following HEMA treatment. Cells (one donor of NOK (A), 3 donors of NOF (B-D) and 2 different OSCC cell lines: CaLH3 (E) and SCC25 (F)) were seeded in 96-well plate in 6 replicates and cultured for 24 hours. The cells were treated with 8mM and 4mM HEMA and examined for cell viability after 6-and 24-hours of treatment. Data were presented as mean ± SD. ANOVA with Tukey’s multiple comparison tests was used for statistical analysis.

Treatment of NOK (NOK1), NOF (NOF1, NOF2 and NOF3) and OSCC cells (CaLH3 and SCC25) with two different concentrations (4 mM and 8 mM) of HEMA led to a significant reduction in cell viability both at 6 h and 24 h in a concentration and time-dependent manner (Figure 1(A–F)). Of note, the degree of reduction in cell variability of CaLH3 (Figure 1(E)) and SCC25 (Figure 1(F)) following HEMA treatment was less than that of NOK (Figure 1(A)) and NOF (Figure 1(B–D)).

### 3.2. HEMA treatment led to an increased number of cleaved Caspase-3 positive cells in NOK cells in 3D-organotypic co-cultures

**Figure 2.**
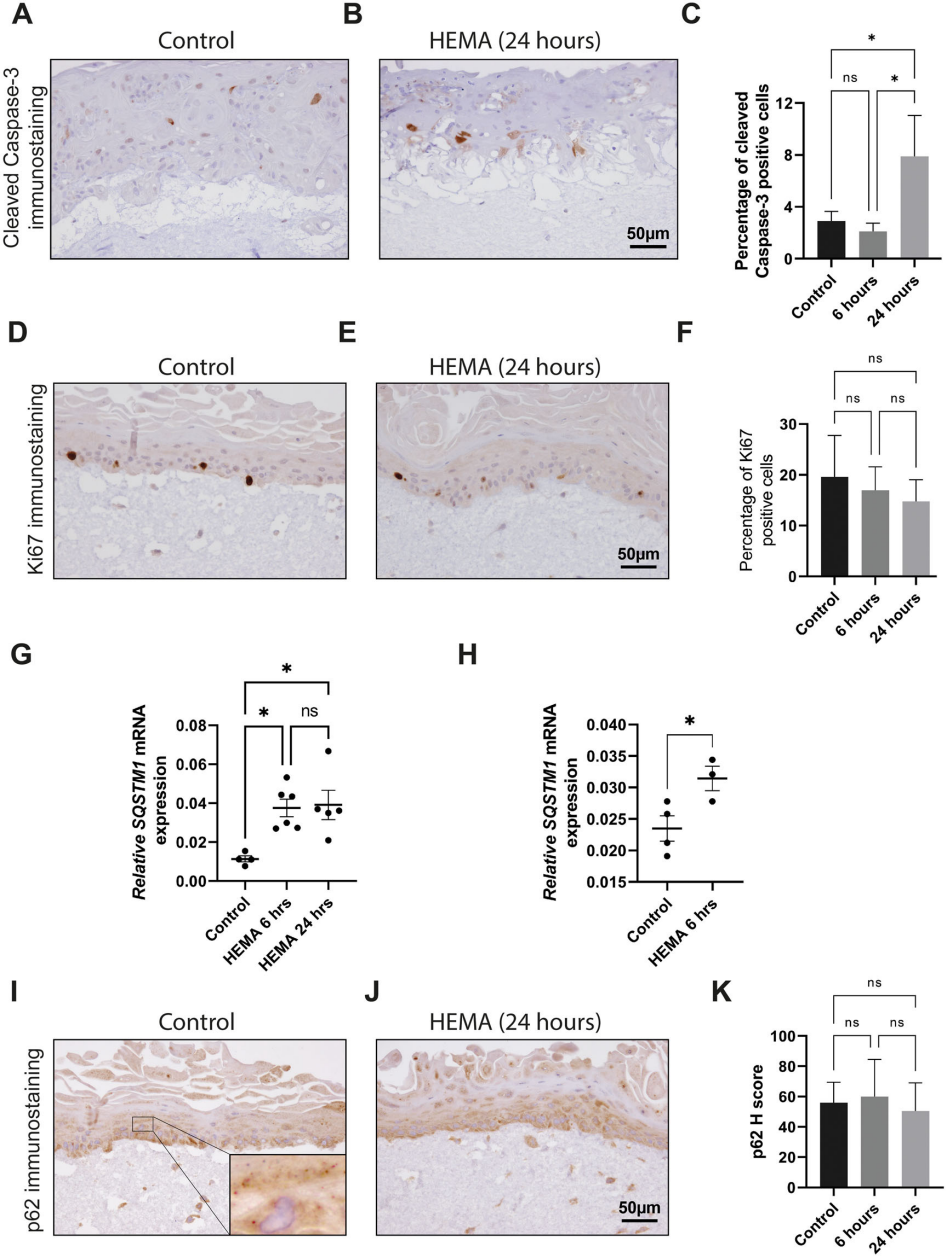
HEMA treatment led to increased apoptosis and upregulation of SQSTM1 mRNA, but not protein in 3D-organotypic co-cultures of NOK cells. Representative images of control and HEMA treated (24 hours) 3D-organotypic co-cultures of NOK showing cleaved caspase-3 positive keratinocytes (A and B). Quantification of immunostaining demonstrated that 4mM HEMA treatment was associated with significantly increased cleaved caspase-3 positive cells in 3D-organotypic co-cultures of NOK (C). 4mM HEMA treatment was not associated with alteration in Ki67 positive cells in 3D-organotypic co-culture of NOK (D-F). A significant upregulation of *SQSTM1* mRNA levels was found in 3D-organotypic co-cultures of NOK cells treated with HEMA both at 6 hours and 24 hours as compared to the untreated controls (G). The upregulation of *SQSTM1* mRNA levels at 6 hours of HEMA treatment was further validated using 3D-organotypic co-cultures consisting of another NOK donor (NOK2) (H). HEMA treatment was not associated with a major change in the immune expression of p62 at both 6 and 24 hours in NOK in 3D-organotypic cultures (I-K). Data were presented as mean ± SD. ANOVA with Tukey’s multiple comparison tests was used for statistical analysis in C, F, G and K. Unpaired Student’s *t*-test was used in H.

To investigate the effect of HEMA treatment (4 mM) on apoptosis, cleaved Caspase-3 immunostaining was performed. Predominantly cytoplasmic cleaved Caspase-3 immunopositivity was observed in scattered epithelial cells mostly in the supra-basal layers of 3D-organotypic co-cultures (Figure 2(A and B)). A significantly higher number of cleaved Caspase-3 immunostaining positive NOK cells was observed following 24 h (7.89% ± 3.1) of HEMA treatment as compared to the untreated control (2.9% ± 0.73) (*p* = 0.018) and HEMA treatment for 6 h (*p* = 0.013) (Figure 2(A–C)). However, there was no significant difference in the number of cleaved Caspase-3 immunostaining positive NOK cells between untreated and 6 h of HEMA treatment (Figure 2(C)).

### 3.3. No significant change in Ki67 immunostaining was found in NOK in 3D-organotypic co-cultures following HEMA treatment

Ki67 immunostaining was performed to examine the effect of HEMA treatment (4 mM) on cell proliferation. Nuclear Ki67 staining was observed mainly in the basal cell layer of the epithelial compartment of 3D-organotypic co-cultures (Figure 2(D and E)). Treatment of 3D-organotypic cultures consisting of NOK1 with HEMA resulted in a slight decrease in nuclear Ki67 immunostaining both at 6 h and 24 h of treatment, though the results were not statistically significant (Figure 2(F)).

### 3.4. HEMA treatment resulted in upregulation of *SQSTM1* mRNA, but not protein, in 3D-organotypic co-cultures of NOK cells

To investigate the effect of HEMA treatment in autophagy flux, *SQSTM1* mRNA/p62 levels were evaluated by RT-PCR and immunostaining, respectively.

A significant upregulation of *SQSTM1* mRNA levels was found in 3D-organotypic co-cultures of NOK cells treated with HEMA both at 6 h (*p* = 0.014) and 24 (*p* = 0.012) hours as compared to the untreated controls (Figure 2(G)). However, there was no significant difference in *SQSTM1* mRNA between 6 and 24 h of treatment (Figure 2(G)). The upregulation of *SQSTM1* mRNA levels at 6 h of HEMA treatment was further validated using 3D-organotypic co-cultures consisting of another NOK donor (NOK2) (Figure 2(H)).

Both NOK and NOF in 3D-organotypic co-cultures were found to express predominantly cytoplasmic p62 with scattered spotty staining (Figure 2(I), inset). In contrast to the upregulation of *SQSTM1* mRNA levels, HEMA treatment of 3D-organotypic cultures consisting of NOK did not lead to a significant alteration in the immune expression of p62 (H score) at both 6 and 24 h (Figure 2(I–K)).

### 3.5. No significant increase in cleaved Caspase-3 positive cells was found in CaLH3 cells in 3D-organotypic co-cultures following HEMA treatment

**Figure 3.**
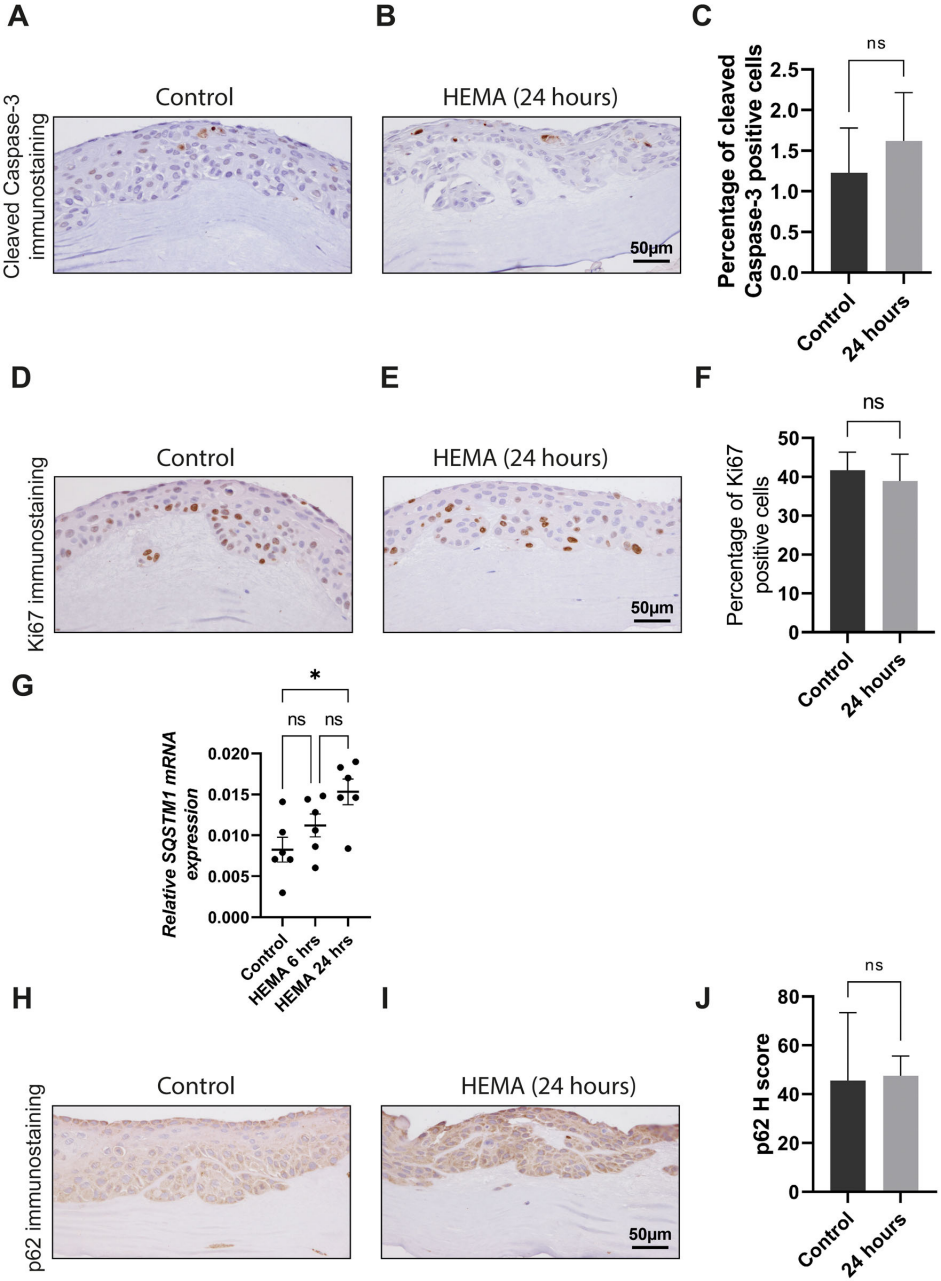
HEMA treatment led to upregulation of *SQSTM1* mRNA, but not protein in 3D-organotypic co-cultures of CaLH3 cells. Representative images of control and HEMA treated (24 hours) 3D-organotypic co-cultures of CaLH3 showing cleaved caspase-3 positive keratinocytes (A and B). Quantification of immunostaining showed that HEMA treatment was associated with a slight but non-significantly increased cleaved caspase-3 positive cells in 3D-organotypic co-cultures (C). HEMA treatment was not associated with alteration in Ki67-positive cells in 3D-organotypic co-cultures (D-E). A significant upregulation of *SQSTM1* mRNA levels was found in 3D-organotypic co-cultures of CaLH3 treated with HEMA at 24 hours as compared to the untreated controls (G). HEMA treatment was not associated with a change in the expression of p62 at 24 hours in CaLH3 in 3D-organotypic cultures (H-J). Data were presented as mean ± SD. Unpaired student’s *t*-test was used for statistical analysis in C, F and J. ANOVA with Tukey’s multiple comparison test was used in G.

Similar to that in 3D-organotypic co-cultures of NOK, cleaved Caspase-3 immunopositivity was observed in scattered epithelial cells mostly in the supra-basal layers (Figure 3(A and B)). Despite an increasing trend for cleaved Caspase-3 immunostaining positive CaLH3 cells following 24 h (1.62% ± 0.59) of HEMA treatment (4 mM), the results were not significantly different from the untreated control (1.2% ± 0.55) (Figure 3(C)).

### 3.6. No significant change in Ki67 immunostaining was found in CaLH3 in 3D-organotypic co-cultures following HEMA treatment

Nuclear Ki67 staining was observed in basal and suprabasal cell layers of the epithelial compartment of 3D-organotypic co-cultures, a feature compatible with the proliferative phenotype of the cancerous oral epithelium (Figure 3(D and E)). Treatment of 3D-organotypic cultures consisting of CaLH3 with HEMA (4 mM) did not significantly alter nuclear Ki67 immunostaining at 24 h of treatment (Figure 3(F)).

### 3.7. HEMA treatment resulted in upregulation of *SQSTM1* mRNA but not of protein levels in 3D-organotypic co-cultures of CaLH3 cells

A significant upregulation of *SQSTM1* mRNA levels was found in 3D-organotypic co-cultures treated with HEMA for 24 h as compared to the untreated controls (*p* = 0.012) (Figure 3(G)). The upregulation *SQSTM1* mRNA at 6 h of treatment was not statistically significant as compared to that of the untreated control (Figure 3(G)). However, no significant change in p62 immunoexpression was observed in 3D-organotypic cultures consisting of CaLH3 following HEMA treatment (4 mM) for 24 h as compared to controls (Figure 3(H–J)).

## 4. Discussion

Besides 2D cultures of primary oral cells (NOK and NOF) and OSCC-cell lines, the current study utilized 3D-organotypic co-cultures mimicking normal and cancerous oral mucosa to investigate the biological effects of HEMA treatment. We demonstrated that HEMA treatment led to reduced viability of NOK, NOF and OSCC-cell lines in 2D culture. The keratinocytes in 3D-organotypic co-cultures of NOK and OSCC-cells (CaLH3) reacted similarly to HEMA treatment, with respect to cell proliferation and activation of autophagy flux. Nevertheless, NOK was found to be more susceptible to apoptosis following HEMA treatment than CaLH3 in 3D-organotypic co-cultures. This difference could be related to the mutual protective interplay between keratinocytes and fibroblasts in 3D-organotypic co-cultures as compared to the 2D culture. Besides this, the multiple layers of keratinocyte and often a protective layer of keratin on top of the 3D-organotypic co-cultures might lead to a lower concentration of HEMA in a deeper layer of cells thereby diluting the biological effects of HEMA in these cells, as compared to the 2D culture where all cells are equally exposed to HEMA.

The reduction in cell viability of NOK, NOF and OSCC-cell lines following HEMA treatment (Figure 1) is in line with previous studies using various cell types including the oral cells [4,5,28]. The reduction in cell viability was found to be time and concentration-dependent. Although not investigated in the current study, the reduction in cell viability could be related to increased apoptosis as suggested previously [4,5]. In the same line, we demonstrated that HEMA treatment for 24 h led to a significant increase in cleaved caspase-3 labelling in NOK in 3D-organotypic co-cultures (Figure 2(C)). These results indicated that HEMA treatment activated the apoptotic program in NOK cells in 3D-organotypic co-cultures. Of note, the apoptotic cells were also slightly, but non-significantly, increased after HEMA treatment in CaLH3 in 3D-organotypic co-cultures as compared to the untreated control (Figure 3(C)). This might be related to the fact that OSCC-cells demonstrate a more aggressive phenotype as compared to the normal oral cells in 2D- and 3D-cultures. Similar results after HEMA treatment were reported in normal and bronchoalveolar carcinoma cells [29]. Our previous studies have linked HEMA-mediated apoptosis to the production of reactive oxygen species and subsequent activation of MAP kinases [3] and DNA damage [5,30,31] in a number of cells. Hence, although we did not investigate the molecular mechanism of apoptosis in the current study, the involvement of the above mechanisms could be important in inducing apoptosis in NOK and CaLH3 cells in 3D-co-cultures.

Besides apoptosis, HEMA treatment was previously linked to disruption in cell cycle progression and reduction in cell proliferation in a number of cells [5,30,32]. However, HEMA treatment was not associated with alteration in Ki67 labelling in NOK and CaLH3 in 3D-organotypic co-cultures (Figures 2(F) and 3(F)). These results indicate that HEMA treatment for 24 h could not affect the proliferation potential of NOK and CaLH3 in 3D-organotypic co-cultures. These observations are in contrast with the previous studies where HEMA treatment was associated with cell-cycle arrest and inhibition of cell proliferation [30,32]. Nevertheless, this could be related to the use of different cell types, different HEMA concentrations and treatment duration, and importantly the response of cells in 2D versus 3D culture. Moreover, it is possible that the keratinocytes and fibroblasts interact and create a mutually protective environment in 3D-organotypic co-cultures, as suggested previously [16].

As one of the response mechanisms against HEMA treatment, cells upregulate genes/proteins involved in the autophagy pathway [13,28]. In the current study, upregulation of *SQSTM1* mRNA levels was found in 3D-organotypic co-cultures of NOK and CaLH3 both at 6 and 24 h of HEMA treatment (Figures 2(G and H) and 3(G)). These results indicate increased autophagy flux following HEMA treatment. Of note, semiquantitative evaluation of immunostaining showed no upregulation of p62 protein in 3D-organotypic co-cultures of NOK and CaLH3 (Figures 2(K) and 3(J)). Despite appearing seemingly contradictory to the increased transcriptional activity of *SQSTM1*, the unchanged expression of p62 might indicate a concomitant increased breakdown of p62 associated with induced autophagy activity following the HEMA treatment [33]. Moreover, our findings underscore the need for further studies that aim to clarify the relationship between HEMA and cell autophagy.

In recent years, microfluidic oral mucosa-on-chip models are gaining popularity for their potential use in the evaluation of toxicity of dental biomaterials. Similar to the 3D-organotypic models, these models have been shown to create stratified epithelium. Such models may provide an additional advantage over 3D-organotypic modes of real-time monitoring of the biological effects of the tested materials [34–36]. However, since the dimensions of the 3D constructs are very small, there have been suggestions that surface effects dominate the volume effect in microfluidic models [37]. Moreover, the necessity for highly specialized instruments limits the practical use of these models [37]. A recent study utilizing a 3D model of oral mucosa consisting of OKF6/TERT2 and gingival fibroblasts demonstrated that HEMA could penetrate the layers of oral keratinocytes and induce cellular oxidative defence response in keratinocytes and fibroblasts [20]. Together with the observation that HEMA treatment led to the induction of *SQSTM1* mRNA levels, 3D-models of oral mucosa appear to display similar biological responses to HEMA as cells in 2D-cultured cells and represent a more clinically relevant model for future HEMA-related studies. Finally, it was noted that the 3D-organotypic co-cultures of NOK and CaLH3 showed similar responses with respect to cell proliferation and autophagic flux, although NOK displayed increased apoptosis compared to CaLH3 cells. Given the challenges in obtaining primary cultures of NOK and problems associated with their rapid differentiation in culture, the possible use of OSCC cells as an alternative to NOK for 3D models represent an area for such future studies.

## Data Availability

The data that support the findings of this study are available from the corresponding author upon reasonable request.
